# Increase in the nuclear localization of PTEN by the *Toxoplasma* GRA16 protein and subsequent induction of p53‐dependent apoptosis and anticancer effect

**DOI:** 10.1111/jcmm.14207

**Published:** 2019-03-04

**Authors:** Sang‐Gyun Kim, Seung‐Hwan Seo, Ji‐Hun Shin, Jung‐Pyo Yang, Sang Hyung Lee, Eun‐Hee Shin

**Affiliations:** ^1^ Department of Parasitology and Tropical Medicine Seoul National University College of Medicine and Institute of Endemic Diseases Seoul Republic of Korea; ^2^ Department of Neurosurgery SMG‐SNU Boramae Medical Center, Seoul National University College of Medicine Seoul Republic of Korea; ^3^ Seoul National University Bundang Hospital Seongnam Republic of Korea

**Keywords:** cell cycle arrest, GRA16‐stable cell line, HAUSP, Hep3B, hepatocellular carcinoma, HepG2, p53, PTEN, *Toxoplasma *GRA16, USP7

## Abstract

This study investigated the efficacy of *Toxoplasma* GRA16, which binds to herpes virus‐associated ubiquitin‐specific protease (HAUSP), in anticancer treatment, and whether the expression of GRA16 in genetically modified hepatocellular carcinoma (HCC) cells (GRA16‐p53‐wild HepG2 and GRA16‐p53‐null Hep3B) regulates PTEN because alterations in phosphatase and tensin homologue (PTEN) and p53 are vital in liver carcinogenesis and the abnormal *p53* gene appears in HCC. For this purpose, we established the GRA16 cell lines using the pBABE retrovirus system, assessed the detailed mechanism of PTEN regulation in vitro and established the anticancer effect in xenograft mice. Our study showed that cell proliferation, antiapoptotic factors, p‐AKT/AKT ratio, cell migration and invasive activity were decreased in GRA16‐stable HepG2 cells. Conversely, the apoptotic factors PTEN and p53 and apoptotic cells were elevated in GRA16‐stable HepG2 cells but not in Hep3B cells. The change in MDM2 was inconspicuous in both HepG2 and Hep3B; however, the PTEN level was remarkably elevated in HepG2 but not in Hep3B. HAUSP‐bound GRA16 preferentially increased p53 stabilization by the nuclear localization of PTEN rather than MDM2‐dependent mechanisms. These molecular changes appeared to correlate with the decreased tumour mass in GRA16‐stable‐HepG2 cell‐xenograft nude mice. This study establishes that GRA16 is a HAUSP inhibitor that targets the nuclear localization of PTEN and induces the anticancer effect in a p53‐dependent manner. The efficacy of GRA16 could be newly highlighted in HCC treatment in a p53‐dependent manner.

## 1 INTRODUCTION

Ubiquitin‐specific peptidases 7/herpes virus‐associated ubiquitin‐specific protease (USP7/HAUSP) is a ubiquitin‐specific protease in human cells that deubiquitinates MDM2 (an E3 ligase), thereby destabilizing p53.^1^, ^2^, ^3^ HAUSP and USP7 are synonymous, and USP7/HAUSP (known as HAUSP) deubiquitinates the tumour‐suppressor phosphatase and tensin homologue (PTEN; deleted in chromosome 10),^1^, ^4^, ^5^ favouring its localization from the nucleus to the cytoplasm and limiting its transcriptional activity, eventually decreasing p53 transcription.^1^, ^4^, ^5^, ^6^ Thus, HAUSP plays a crucial role in cell proliferation and differentiation by deubiquitinating MDM2 and PTEN; HAUSP suppression is vital for anticancer therapeutic intervention, simultaneously focusing on HAUSP as a candidate druggable target.^1^, ^2^, ^3^, ^4^, ^5^, ^6^

Herpes virus‐associated ubiquitin‐specific protease is overexpressed in various human cancer types, including prostate cancer, multiple myeloma tumours, neuroblastoma, epithelial ovarian cancer, non‐small cell lung cancer, breast cancer, hepatocellular carcinoma (HCC) and lymphocytic leukaemia.^1^, ^2^, ^3^, ^6^, ^7^, ^8^ Conversely, the negative regulation of HAUSP exhibits a crucial clinical significance for estimating the prognosis of tumour suppression.^5^, ^7^ Thus, identification of HAUSP inhibitors is imperative for the therapeutic intervention of cancer. Based on the findings of a recent study,^9^ the authors probed whether the properties of GRA16 recognized as a new HAUSP inhibitor could be available for anticancer intervention because the precise mechanism of GRA16 in inducing an anticancer effect remains unclear to date.

*Toxoplasma gondii* (*T gondii*) is an intracellular parasite that infects multiple organs and tissues in acute infection and the brain in chronic infection^9^, ^10^ and regulates the host immunity for its survival during infection.^10^, ^11^ Briefly, the immunomodulatory activities of *T gondii* are mediated by infection as well as several *T gondii*‐specific molecules such as rhoptry proteins (ROP), dense granule proteins (GRA), *T gondii* profilin‐like protein (TgPLP) and the lysate antigenic proteins.^9^, ^10^ Thus, our objective was to determine the intermediate events between HAUSP inhibition and p53 stabilization and also the anticancer effect. In particular, p53 transcriptional activity is often disrupted in HCC by highly expressed HAUSP; moreover, the expression of nuclear PTEN decreases in patients with advanced‐stage HCC.^5^, ^7^, ^17^, ^18^ Thus, HCC forms an appropriate model for our study; indeed, it has been known that HCC is one of the 10 most common cancer types worldwide with no ideal treatment.^17^

Thus, this study aimed to investigate transcriptional gene expressions associated with PTEN and subsequent apoptosis after HAUSP inhibition by GRA16. Furthermore, it investigated the characteristics of molecular networks primarily associated with nuclear PTEN changes between HAUSP and p53 in GRA16‐stable cells.

## 2 MATERIALS AND METHODS

### 2.1 Cell culture

We purchased HepG2 and Hep3B cells, human liver cancer cell lines, from the Korean Cell Line Bank (Seoul, Korea) and cultured with Dulbecco's Modified Eagle's Medium (DMEM; 1×, liquid (high glucose); WELGENE Inc, Gyeongsan, Korea] containing 10% foetal bovine serum (WELGENE Inc) and 1% antibiotic antimycotic solution (WELGENE Inc) in 100‐mm dishes (SPL Life Sciences, Pocheon, Korea) under 5% CO_2_ and 37ºC in a CO_2_ incubator.

### 2.2 Plasmid construction for preparing GRA16‐gene stable cell line

The *T gondii*‐derived dense granule protein 16 (TgGRA16) was prepared by gene cloning for the preparation of a stable cell line that continuously expressed the *GRA16* gene. Furthermore, the *GRA16* gene was amplified by PCR with a pair of specific primers (Table 1) designed in accordance with the reference sequence from the ToxoDB database (Gene ID: ToxoDB, TGGT1_208830). Then, the products (1518 bp) were inserted into pBABE‐HA vectors (Addgene, Cambridge, MA, USA) digested with *Eco*RI and *Sal*I and confirmed by the gene sequence (Cosmo Genetech Co Ltd., Seoul, Korea). Typically, retrovirus vectors lack viral structural genes and require packaging cells to generate viral articles. Thus, Platinum‐A packaging cells (Cell Biolabs, Inc, San Diego, CA) were conventionally used to establish stable producers of recombinant retroviruses. Furthermore, Platinum‐A cells were transfected with pBABE HA II‐GRA16 (GRA16‐inserted vector, pBABE‐GRA16) or pBABE HA II‐empty (empty vector, pBABE‐empty) using the Lipofectamine 3000 Transfection Kit (Life Technologies, NY, USA) and Opti‐MEM media (Life Technologies, Gaithersburg, MD). Then, the recombinant retroviruses were infected to HepG2 or Hep3B for 48 hours to prepare stable cell lines with pBABE‐GRA16 or pBABE‐empty in a human HCC cell line, HepG2 or Hep3B. Retrovirus‐transfected cells were then selected as puromycin (2 mg/mL)‐resistant stable cell lines.

**Table 1:** Primer sequences

Gene	Forward primer (5′‐3′)	Reverse primer (5′‐3′)
*GRA16*	5′‐ATG TAT CGA AAC CAC TCA GGG‐3′	5′‐TCA CAT CTG ATC ATT TTT CCG C‐3′
*pBABE*	5′‐CTC TTG ACA TTG CTC AGA CCT GT‐3′	5′‐AAG TTG CAG GAC CAC TTC TG‐3′
*Foxo3a*	5′‐TGG ATG CGT GGA CGG ACT TC‐3′	5′‐CGT GCA CGG CTT GCT TAC TG‐3′
*P21*	5′‐TCC TCA TCC CGT GTT CTC CT‐3′	5′‐CAC CCT GCC CAA CCT TAG AG‐3′
*BAX*	5′‐CTT TTG CTT CAG GGT TTC ATC CAG G‐3′	5′‐ATC CTC TGC AGC TCC ATG TTA CTG‐3′
*Bcl‐2*	5′‐ACT GAG GAG CTT TGT TTC AAC CAA G‐3′	5′‐GCC ACG TAA AGC AAC TCT CTA AAG G‐3′
*Survivin*	5′‐AGT CCC TGG CTC CTC TAC TG‐3′	5′‐TGA AGG TTG GGC TGA CAG AC‐3′
*MDM2*	5′‐AGG AAT CAT CGG ACT CAG GTA CAT C‐3′	5′‐CAG ATT TGT GGC GTT TTC TTT GTC G‐3′
*MMP2*	5′‐AGC ATG TCC CTA CCG AGT CT‐3′	5′‐AAA CAG ATG GCA AAC ACG GC‐3′
*PTK2*	5′‐TGA TGC ATG GTG TGA AGC CT‐3′	5′‐CCA GGA TTG TGC TGA GCT GA‐3′
*PTEN*	5′‐CCA GTC AGA GGC GCT ATG TG‐3′	5′‐TCG TGT GGG TCC TGA ATT GG‐3′
*GAPDH*	5′‐GGT GAA GGTC GGA GTC AAC GGA‐3′	5′‐GAG GGA TCT CGC TCC TGG AAG A‐3′


### 2.3 Co‐immunoprecipitation for binding between GRA16 and USP7/HAUSP

For the co‐immunoprecipitation (co‐IP) assay, total proteins in 5 × 10^6^ HepG2 or Hep3B cells were extracted by incubating for 15 minutes at room temperature (RT) using 100‐μL mammalian protein extraction reagent (M‐PER; Pierce Biotechnology, Inc, Rockford, IL, USA). Protein A/G‐plus agarose beads previously reacted with HA‐Tag Ab were added in 0.5 mg of each extracted protein (HepG2 or Hep3B) and incubated for 4 hours at 4°C. The supernatant of each sample was analysed by Western blotting with the HAUSP Ab (Cell Signaling Technology, Danvers, MA, USA).

### 2.4 Cell proliferation

A total of 5 × 10^3^ cells were seeded on a 96‐well plate and incubated for 2, 4 and 6 days to assess the cytotoxicity of cells that were divided into experimental groups (control, vector, GRA16) in HepG2 and Hep3B cells. Cell viability at each incubation time was assessed using the Cell Counting Kit (CCK‐8; Dojindo, Kumamoto, Japan) and measured in terms of optical density at 451 nm using a microplate reader (Thermo Fisher Scientific, Waltham, MA, USA). For the cell proliferation analysis, HepG2 and Hep3B cells were seeded into 24‐well plates at 3 × 10^4 ^cells/well and cultured for 6 days. The degree of cell proliferation was monitored by the Trypan blue exclusion test using a haemocytometer on days 2, 4 and 6 after cell seeding. All experiments were performed in triplicates, and results were obtained through three independent experiments for each study.

### 2.5 Gene expression of GRA16 for HepG2‐GRA16 and Hep3B‐GRA16 cell lines

Total RNA was extracted using the HiGene Total RNA Prep Kit (BIOFACT, Daejeon, Korea) and reverse‐transcribed to cDNA using the Reverse‐Transcription Master Premix Kit with oligo d(T)15 primer (ELPIS Biotech, Daejeon, Korea). Then, cDNA samples were subjected to PCR to validate the gene expression of GRA16 in stable cell lines. The primers were designed by referring to the sequence from the ToxoDB database (Gene ID: ToxoDB, TGGT1_208830) using the Geneious Pro R8 program (Biomatters Ltd., Auckland, New Zealand); the primer sequences are presented in Table 1.

### 2.6 Real‐time PCR

Real‐time PCR was performed to target genes using the CFX96 Real‐Time PCR Detection System (Bio‐Rad Laboratories, Hercules, CA) and SYBR Green I detection chemistry (Bio‐Rad Laboratories). Primer sequences are presented in Table 1. The data were analysed using the Bio‐Rad CFX manager software ver 3.1 (Bio‐Rad Laboratories). All cDNA samples were assessed by the fold change in the target gene expression compared with the control and, then, calculated for the fold change in the gene expression for vector (transfected by empty vector) or GRA16 (stable cells with GRA16) compared with the control in each target gene.

### 2.7 Western blotting

Cells were cultured in a 6‐well plate with complete DMEM medium for 6 days, and total proteins were extracted from cells using the M‐PER Mammalian Protein Extraction Kit (Pierce Biotechnology, Inc). Proteins (50 μg) were separated using SDS‐PAGE and transferred to an Immun‐Blot PVDF membrane (Bio‐Rad Laboratories). The membrane was subsequently incubated with anti‐p53 Ab (Santa Cruz Biotechnology), anti‐PTEN Ab (Santa Cruz Biotechnology), anti‐AKT Ab (Enzo Life Sciences, Farmingdale, NY), anti‐phospho‐AKT (Ser473) Ab (Enzo Life Sciences) and anti‐‐actin Ab (Santa Cruz Biotechnology) overnight at 4°C, and then stained with antirabbit IgG‐ or antimouse IgG‐peroxidase conjugate as a secondary Ab (Santa Cruz Biotechnology). Then, to detect PTEN expression in the nucleus and cytoplasm of HepG2 and Hep3B cells, the cultured cells were divided into nuclear and cytoplasmic extracts using NE‐PER nuclear and cytoplasmic extraction reagents (Pierce Biotechnology, Inc), and the extracted proteins (25 μg) were analysed by SDS‐PAGE and Western blotting with an anti‐PTEN (Santa Cruz Biotechnology), anti‐lamin B (M‐20; Santa Cruz Biotechnology) and anti‐‐actin Ab (Santa Cruz Biotechnology). Signals were detected by exposing the membrane to the enhanced chemiluminescence (ECL) Pierce kit (Pierce Biotechnology, Inc) using the Fuji LAS 1000 Lumino Image Analyzer (Fujifilm Corporation, Tokyo, Japan) and evaluated using an image calculator (ImageJ program).

### 2.8 Immunofluorescence for the GRA16 expression and PTEN nuclear localization

Immunofluorescence images of cells were obtained after immunostaining with HA‐Tag Ab (Elabscience) and anti‐PTEN Ab (Santa Cruz Biotechnology) to investigate GRA16 expression in HepG2‐ and Hep3B‐GRA16 cells and the nuclear localization of PTEN. The immunostained cells were observed using fluorescent microscopy (Leica DMI6000 B).

### 2.9 Cell cycle analysis

HepG2 and Hep3B cells with and without GRA16 were seeded at 5 × 10^5^ cells/well in a 6‐well plate for 6 days and stained with propidium iodide (PI). Results were analysed by flow cytometry using FACSCalibur (Becton Dickinson, San Jose, CA). Data were acquired by the linear amplification of FL2 and analysed for cell proportions in the G_0_‐G_1_, S and G_2_‐M phases of the cell cycle using the CellQuest software (Becton Dickinson).

### 2.10 Cell apoptosis assays using flow cytometry

HepG2 and Hep3B cells were cultured at 5 × 10^5^ cells/well in 6‐well plates for 6 days and harvested by trypsin‐EDTA treatment. The cell pellets were resuspended in 500‐μL Annexin V binding buffer, followed by staining with both 5 μL of Annexin V‐APC (BioLegend, San Diego, CA) and 2‐μL PI (1 mg/mL in distilled water; Sigma). After 15‐minute incubation, apoptotic cells were analysed by flow cytometry using the FACSCalibur (Becton Dickinson).

### 2.11 Wound healing assay

HepG2 and Hep3B cells with and without GRA16 were seeded at 5 × 10^5^ cells/well in a 24‐well plate and incubated for 24 hours in the CO_2_ incubator. At approximately 90% confluency, the inner bottom of the well was scratched by drawing a line with a 200‐μL Eppendorf yellow tip, and the movement of cells into the scratched space was confirmed at 0, 24 and 48 hours by microscopic images using a digital camera (Leica DFC 280; Leica Microsystems, Bensheim, Germany) and a microscope (BX‐51; Olympus Corporation) with the AF6000 Leica Las‐X software (Leica Microsystems). Based on the wound width, data were evaluated by the migration distance using the ImageJ program.

### 2.12 Transwell migration/invasion assay

The migration and invasion ability of cells were assessed using the Matrigel‐coated Transwell cell culture chambers (8‐μm pore size; Millipore, Billerica, MA, USA). Briefly, 700‐μL complete DMEM medium was added to the lower chamber in a 24‐well plate. Then, cells to be placed on the top were resuspended in the serum‐free DMEM medium, and 200 μL of the resuspended cells solution (1 × 10^5^ cells) was seeded into the upper chamber of each Transwell. After 24‐hour incubation, the cells were fixed with 4% formaldehyde for 10 min at RT and permeabilized by 100% methanol for 2 minutes. After washing with PBS, cells were stained with 10% Giemsa staining solution for 15 minutes at RT. Finally, after noninvasive cells were scraped off with a cotton swab, invasive cells were counted in five randomly selected areas per well (magnification, ×200). Data were obtained in triplicates.

### 2.13 Ethics statement

All animal experiments were approved by the Institutional Animal Care and Use Committee in Seoul National University (SNU‐170717‐2), and animals were maintained in the facility following standards of the Animal Protection Act and the Laboratory Animal Act in Korea. All experiments were conducted according to global standards such as those of AAALAC, and all efforts were made to ensure minimal animal suffering (SNUIBC‐R180523‐1).

### 2.14 Cancer cell xenograft and tumour production in GRA16 stable cell lines

Five‐week‐old BALB/c nude mice were purchased from Orient Bio Inc (Seongnam, Korea) and housed at RT with a 12‐h light‐dark cycle in a specific pathogen‐free barrier zone of the animal facilities at Seoul National University College of Medicine. After 1‐week acclimatization before the experiment, mice were categorized into experimental groups by weight constantly. To induce tumour mass formation, HepG2 and Hep3B cells (3 × 10^6^ cells/100‐μL PBS) were mixed with 100‐μL Matrigel Basement Membrane Matrix, Phenol‐Red free (BD Biosciences) and subcutaneously injected into the right side of the waist of each mouse. Tumour sizes were measured once every 3 days after visible tumour formation in each group of mice injected with HepG2 ((n = 5; control, vector and GRA16‐group) and Hep3B (n = 5 (control) and n = 6 (vector and GRA16 group)) during the experimental period and were calculated using the formula: *V *= *ab* ^2^/*2* (where *a* and *b* are tumour length and width respectively).

### 2.15 Statistical analysis

All statistical analyses were performed using the GraphPad Prism 5 software (GraphPad, La Jolla, CA, USA). Data are presented as mean ± standard deviation (SD) of three independent experiments, each performed in triplicates. One‐way analysis of variance (ANOVA) was performed followed by the Tukey's multiple comparison test to assess the differences between the experimental groups. Two‐way ANOVA followed by the Bonferroni's post hoc comparisons test was used to assess differences between the experimental groups. *P* < 0.05 was considered statistically significant.

## 3 RESULTS

### 3.1 Changes in the cell proliferation and total cell counts after establishing a GRA16‐expressing stable cell line for HepG2 and Hep3B

**Figure 1:**
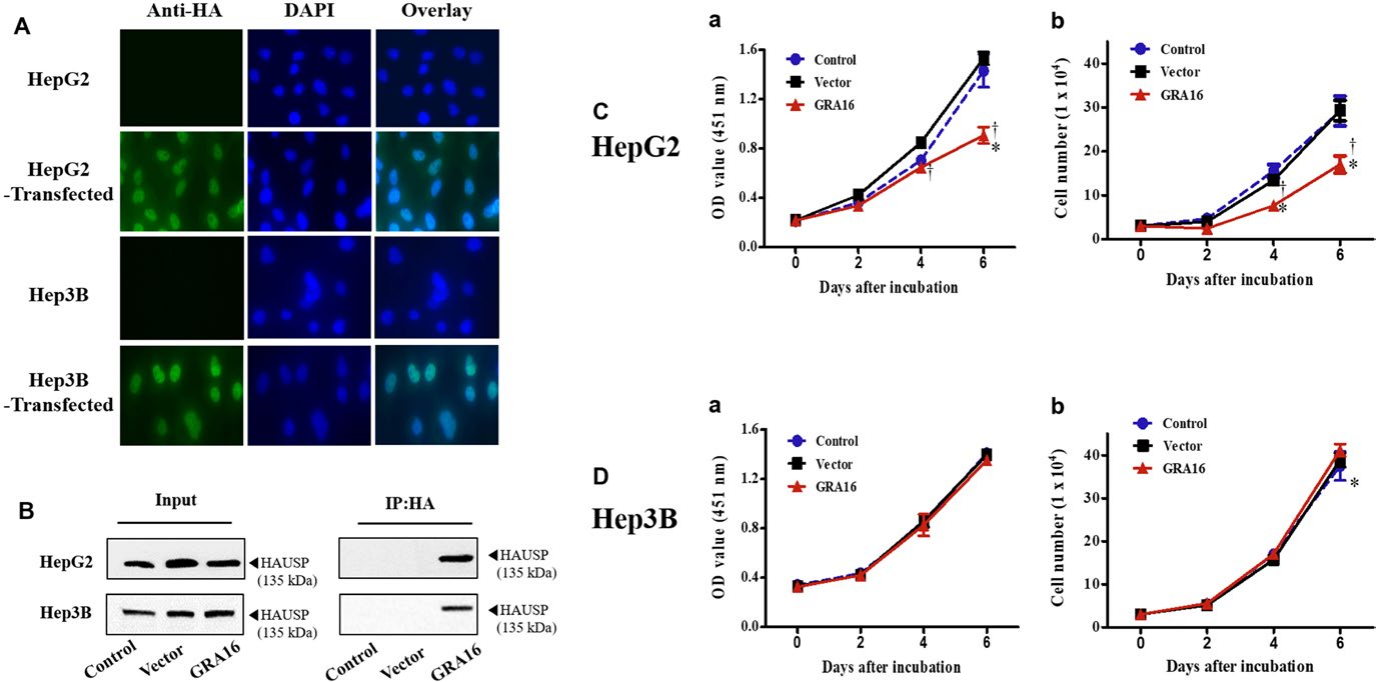
Cell proliferation and total cell counts after establishing a GRA16‐stable cell line for HepG2 and Hep3B. A, The expression of HA‐tagged GRA16 proteins in the immunofluorescence assay using anti‐HA antibody. B, GRA16 protein bound to HAUSP within GRA16‐stable cells (Co‐IP analysis). C, The cell proliferation and total cell number measured by using the Cell Counting Kit (OD value at 451 nm) and the Trypan blue exclusion test (cell number) on days 2, 4 and 6 after incubation. Data are presented as the mean ± SD. *The significant difference in the vector and GRA groups compared with control (*P* < 0.05). †The significant difference between vector and GRA16 stable cells (*P* < 0.05)

A stable cell line expressing the GRA16 protein for HepG2 and Hep3B, which are p53‐wild‐ and p53‐null type, respectively, as human HCC cells was established to determine the role of GRA16 as an HAUSP inhibitor in cancer cells (Figure 1). All experimental groups were divided into control (no transfection), vector (transfected with pBABE vector) and GRA16 (transfected with pBABE‐GRA16 vector). We confirmed GRA16 expression in the stable cell line by intracellular GRA16 expression using green fluorescent protein (GFP)‐immunostaining (Figure 1A) and co‐immunoprecipitation (co‐IP) assay results between GRA16 and HAUSP (Figure 1B). As shown in Figure 1A, GRA16 was stably expressed in cells of both GRA16‐stable HepG2 and Hep3B. In particular, the GRA16 protein was present in the GRA16‐HAUSP complex in GRA16‐stable cells (HepG2 and Hep3B) (Figure 1B). These findings confirmed the establishment of GRA16‐expressing stable cell lines and that GRA16 is presented by binding to HAUSP intracellularly. After transfections, cell proliferation and total cell counts in the control and vector groups increased gradually during 6 days with no differences observed in both HepG2 and Hep3B (Figure 1C,D). However, the cell count of GRA16‐stable HepG2 cells had significantly decreased with a significant decrease in cell proliferation in comparison to the control and vector groups (*P* < 0.05; Figure 1C‐a and C‐b), whereas cell proliferation of GRA16‐stable Hep3B cells increased continuously, with no difference in the total cell counts in the control and vector groups (Figure 1C). Although the reduction in the total cell number during the 6‐day cell culture was apparent in GRA16‐stable HepG2 cells, it was not observed in GRA16‐stable Hep3B cells (Figure 1D‐a and D‐b).

### 3.2 Protein expressions of PTEN and p53 as well as AKT phosphorylation in GRA16‐stable‐HepG2 and ‐Hep3B cells

**Figure 2:**
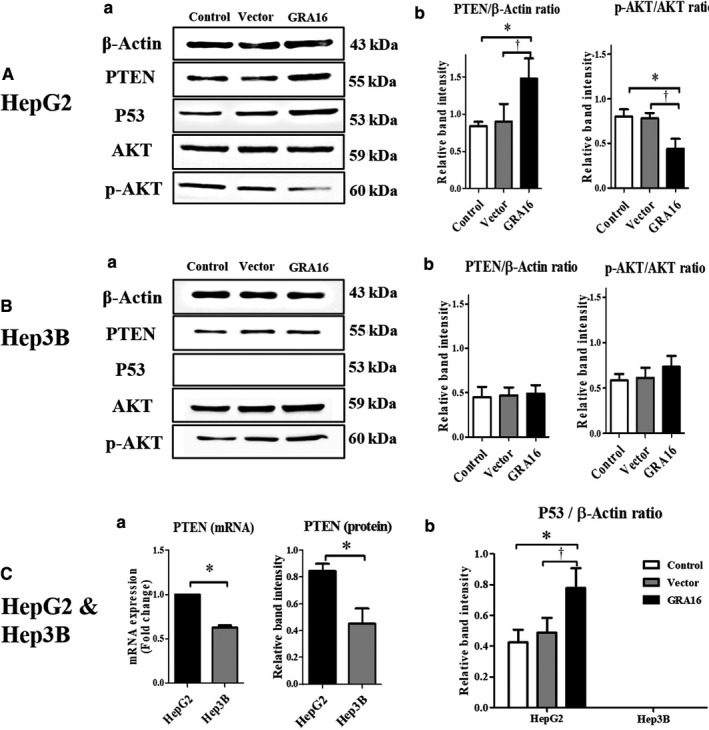
Protein expressions of PTEN and p53, as well as the AKT phosphorylation, in GRA16‐stable‐HepG2 and ‐Hep3B cells. (A‐a) and (B‐a) Western blotting results on PTEN, p53 and phosphorylation of AKT in HepG2 and Hep3B cells. (A‐b) and (B‐b) the relative band intensity of PTEN/β‐actin and p‐AKT/AKT in HepG2 and Hep3B. (C‐a) Endogenously expressed PTEN levels (mRNA and protein) in the control group (HepG2 or Hep3B). (C‐b) mRNA level of p53 normalized by β‐actin in HepG2 and Hep3B. *The significant difference between the control and GRA16 groups (*P* < 0.05). †The significant difference between the vector and GRA groups (*P* < 0.05)

p53 protein expression was observed in p53‐wild HepG2 cells but not in p53‐null Hep3B cells (Figure 2A‐a and B‐a). Although PTEN expression was significantly increased, AKT phosphorylation was significantly decreased in GRA16‐stable HepG2 cells in comparison to the control and vector groups (*P* < 0.05; Figure 2A‐b). However, PTEN expression in Hep3B cells was lower originally than that in HepG2 cells, and the p‐AKT/AKT ratio in Hep3B cells exhibited no difference in comparison to control and vector groups (Figure 2B‐b). We examined the mRNA level of PTEN between HepG2 and Hep3B to confirm whether the protein expression of PTEN in control‐Hep3B cells is lowered than that in control‐HepG2 cells (*P* < 0.05; Figure 2C‐a). In addition, the mRNA level was lowered in Hep3B in comparison to HepG2 (*P* < 0.05; Figure 2C‐a). These findings suggested that regulation of PTEN expression and AKT phosphorylation is closely associated with endogenous p53 expression. Thus, we anticipated that GRA16 as a HAUSP inhibitor plays a role in the up‐regulation of p53 by increasing the expression of PTEN protein in p53‐wild‐type HepG2. As anticipated, the relative intensity of the p53 protein normalized by β‐actin was significantly increased in GRA‐stable HepG2 cells in comparison to the control and vector groups (*P* < 0.05; Figure 2C‐b). However, because the p53‐null‐type Hep3B is absent in p53 expression, further regulation of PTEN expression and AKT phosphorylation was not observed in this study (Figure 2B‐b and C‐b). Accordingly, these findings suggested that in the presence of endogenous p53, GRA16 as a HAUSP inhibitor increases PTEN expression and simultaneously decreases AKT phosphorylation, subsequently increasing p53 expression. In the absence of p53, the role of GRA16 is so limited that it cannot increase PTEN expression and p53 stabilization.

### 3.3 An increase in the nuclear localization of PTEN in GRA16‐stable‐HepG2 cells

**Figure 3:**
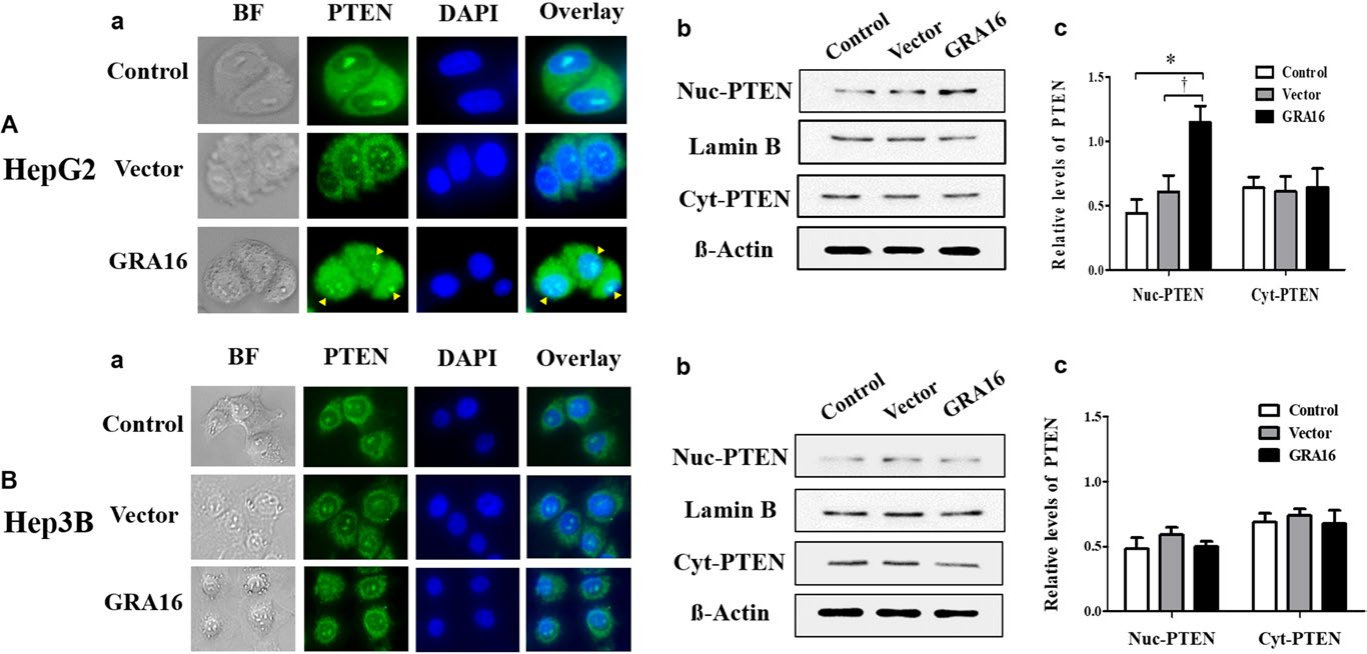
The increase in the nuclear localization of PTEN in GRA16‐stable‐HepG2 cells. (A‐a) and (B‐a) Fluorescent intensity with which the PTEN protein expressed within cells was immunostained. BF; bright field; PTEN, GFP‐positive green fluorescence using anti‐PTEN Ab; DAPI, nuclear DNA stained with DAPI; Overlay, the merged fluorescent image (overlaid between GFP and DAPI fluorescence). (A‐b) and (B‐b) Western blot images for nuclear PTEN (Nuc‐PTEN) and cytoplasmic PTEN (Cyt‐PTEN). Protein bands of Lamin B and β‐actin show the expression of housekeeping proteins in the nucleus and cytoplasm respectively. (A‐c) and (B‐c) Relative levels of PTEN, which are normalized by Lamin B and β‐actin, respectively, in the nucleus and cytoplasm in HepG2 and Hep3B. *The significant difference between the control and GRA16 groups (*P* < 0.05). †The significant difference between the vector and GRA groups (*P* < 0.05)

Based on the results presented above, indicating that the increase in PTEN and p53 expressions was distinct in GRA16‐stable‐HepG2 cells, we anticipated an increase in the nuclear localization of PTEN by GRA16. Accordingly, we confirmed the presence of nuclear PTEN using the immunofluorescence assay and investigated the differences in protein concentrations of the nuclear PTEN and cytosol PTEN using Western blotting (Figure 3). In addition, anti‐PTEN Ab stained the PTEN protein in the nucleus and cytoplasm of GRA16‐stable‐HepG2 and ‐Hep3B cells (Figure 3A‐a and B‐a). The relative intensity of fluorescence in the nucleus of HepG2 cells was higher in the GRA16 group than that in the control and vector groups (Figure 3A‐a). However, the intensity of fluorescence in the nucleus of Hep3B cells was equivalent to that in the control and vector groups (Figure 3B‐a). We investigated the relative level of PTEN expressed in the nucleus and cytoplasm using Western blotting to demonstrate an increase in nuclear PTEN in GRA16‐stable cells (Figure 3A‐b and B‐b). We determined the relative levels of PTEN by the ratio of Lamin B in the nucleus and the ratio of β‐actin in the cytoplasm (Figure 3A‐c and B‐c). The nuclear PTEN level was significantly increased in GRA16‐stable‐HepG2 cells than that in the control and vector groups (*P* < 0.05; Figure 3A‐c). In contrast, the cytoplasmic PTEN level remained unchanged regardless of the presence of the GRA16 protein (Figure 3A‐c). Furthermore, the levels of nuclear and cytoplasmic PTEN in Hep3B cells were not affected by the presence of GRA16 (Figure 3B‐c). These findings strongly suggested that the role of GRA16 as a HASUP inhibitor increased the nuclear localization of PTEN in the presence of endogenous p53.

### 3.4 The retained cell cycle of the G_2_‐M phase in GRA16‐stable‐HepG2

**Figure 4:**
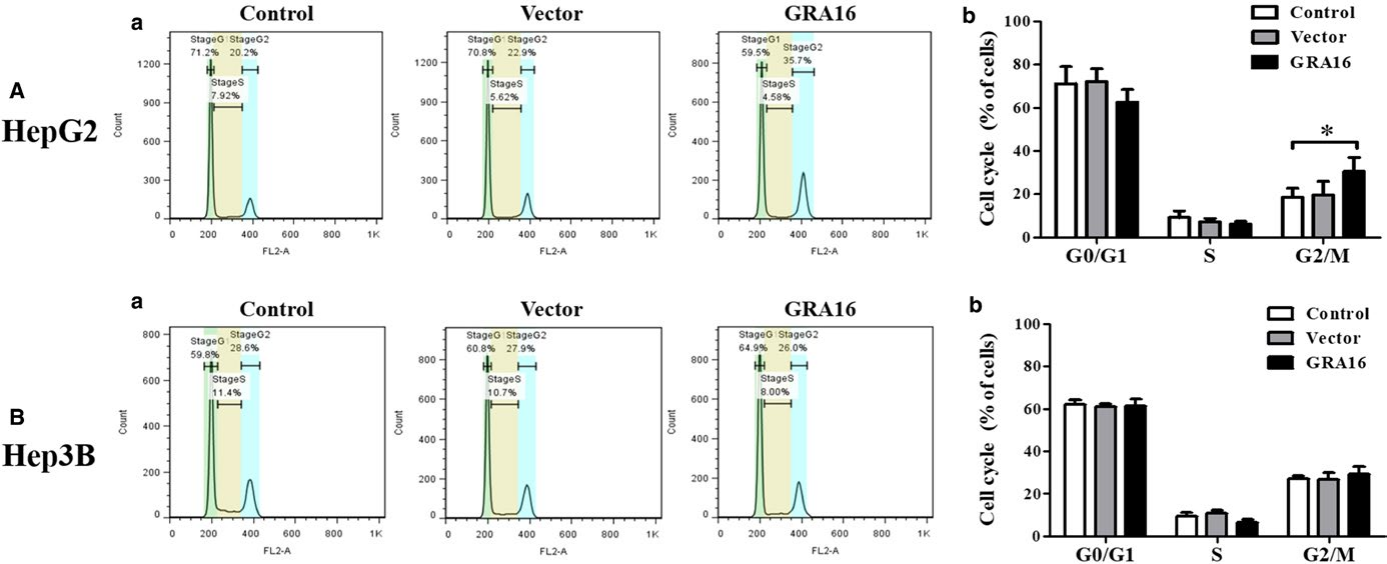
Regulation of the cell cycle by GRA16 in HepG2 and Hep3B. (A‐a) and (B‐a) Images for the pattern of cell cycle analysed by the FACS using the PI staining. (A‐b) and (B‐b) The figures show the difference in relative fractions of the cell cycle (%) in HepG2 and Hep3B. *The significant difference between the control and GRA groups in each fraction (*P* < 0.05)

The results presented above, which demonstrate a decrease in the total cell number and AKT phosphorylation as well as an increase in cell apoptotic factors and nuclear PTEN in GRA16‐stable‐HepG2 cells, implied subsequent changes in cell cycles (Figure 4). Thus, we quantified the percentage of cells occupied during each phase of the cell cycle using the FACS assay. Figure 4A‐a and B‐a presents images for the results of the cell cycle analysis by the FACS assay, and Figure 4A‐b and B‐b presents the quantified results in each phase of the cell cycle. In this study, GRA16‐stable‐HepG2 cells exhibited a significant retention in the G_2_‐M phase in comparison to the control cells (*P* < 0.05; Figure 4A‐b). However, GRA16‐stable‐Hep3B cells did not demonstrate any difference with other groups (control and vector) in (%) of cells occupied in each cell cycle (Figure 4B‐b). Table 2 presents these findings in detail.

**Table 2:** Percentage (%) of cells occupied in each phase of the cell cycle in HepG2 and Hep3B

Cells	Group	G0/G1 (%)	S (%)	G2/M (%)
HepG2	Control	71.3 ± 6.41	9.2 ± 2.51	18.5 ± 3.47
	Vector	72.2 ± 4.80	7.3 ± 1.20	19.7 ± 5.10
	GRA16	62.7 ± 4.76	6.1 ± 1.09	30.6 ± 5.28
Hep3B	Control	61.9 ± 1.80	9.4 ± 1.48	26.9 ± 1.32
	Vector	61.0 ± 1.07	10.9 ± 1.11	26.7 ± 2.47
	GRA16	61.2 ± 2.72	6.6 ± 1.23	29.1 ± 2.98

### 3.5 Differences in inductions of apoptosis and p53‐related apoptotic factors between GRA16‐stable‐HepG2 and ‐Hep3B cells

**Figure 5:**
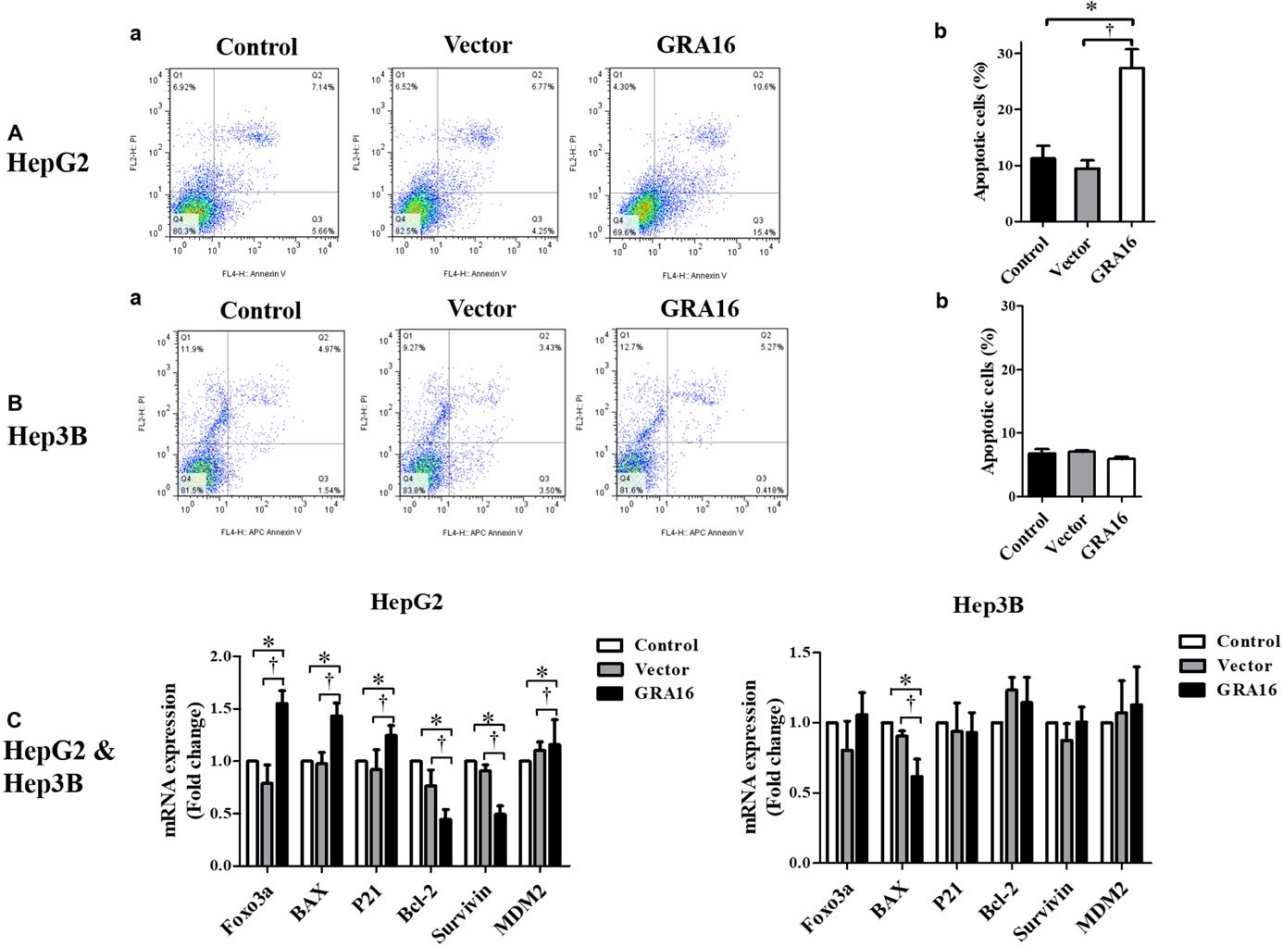
Inductions of apoptosis and p53‐related apoptotic factors in GRA16‐stable‐HepG2 cells. (A‐a) and (B‐a) Percentages of apoptotic cells analysed by the Annexin V and PI staining. (A‐b and B‐b) Apoptotic cells (%) were analysed by FACS and compared among the control, vector and GRA16 groups. C, mRNA expressions in Foxo3a, BAX and p21 for apoptotic factors as well as in Bcl‐2, Survivin and MDM2 for antiapoptotic factors. *The significant difference between the control and GRA groups (*P* < 0.05). †The significant difference between the vector and GRA groups (*P* < 0.05)

To investigate p53‐dependent cell apoptosis in GRA16‐stable HepG2 cells, we assessed the effect of GRA16 on the induction of cell apoptosis between GRA16‐stable‐HepG2 and ‐Hep3B cells using the FACS method with double staining of Annexin V and propidium iodide (Figure 5); the result is summarized by the percentage of total apoptotic cells, including the early and late stage of apoptosis (Figure 5A‐b and B‐b). The findings revealed that an increase in apoptotic cells under the presence of GRA16 was observed in HepG2 but not Hep3B in comparison to the control and vector groups (*P* < 0.05; Figure 5A‐b and B‐b). This finding offers a crucial point to elucidate the role of GRA16 as a HAUSP inhibitor, that is, GRA16 plays a role in the interaction between PTEN and p53 in the process from HASUP inhibition to p53 stabilization, and as a precondition for that role, the presence of undamaged p53 is necessary for the apoptosis of cancer cells by the role of GRA16. For the apoptosis related with p53 in GRA16‐stable HepG2 cell, we validated mRNA expressions for apoptotic (Foxo3a, BAX and P21) and antiapoptotic factors (Bcl‐2, Survivin and MDM2) associated with the p53 function (Figure 5C). As anticipated, the mRNA levels of apoptotic factors (Foxo3a, BAX and P21) in GRA16‐stable HepG2 cells were significantly increased in comparison to both control and vector groups (*P* < 0.05; Figure 5C). In contrast, the mRNA levels of antiapoptotic factors (Bcl‐2 and Survivin) were decreased in GRA16‐stable HepG2 cells (*P* < 0.05; Figure 5C). However, the increase in apoptotic factors was not observed in Hep3B cells (Figure 5C). Meanwhile, antiapoptotic factors exhibited no change in GRA16‐stable Hep3B cells as well as the control and vector groups (Figure 5C). These findings implied that the role of GRA16 as a HASUP inhibitor was exhibited in p53‐wild‐type cells.

### 3.6 Effects of GRA16 on the mobility and invasive activity of GRA16‐stable‐HepG2 and ‐Hep3B cancer cells

**Figure 6:**
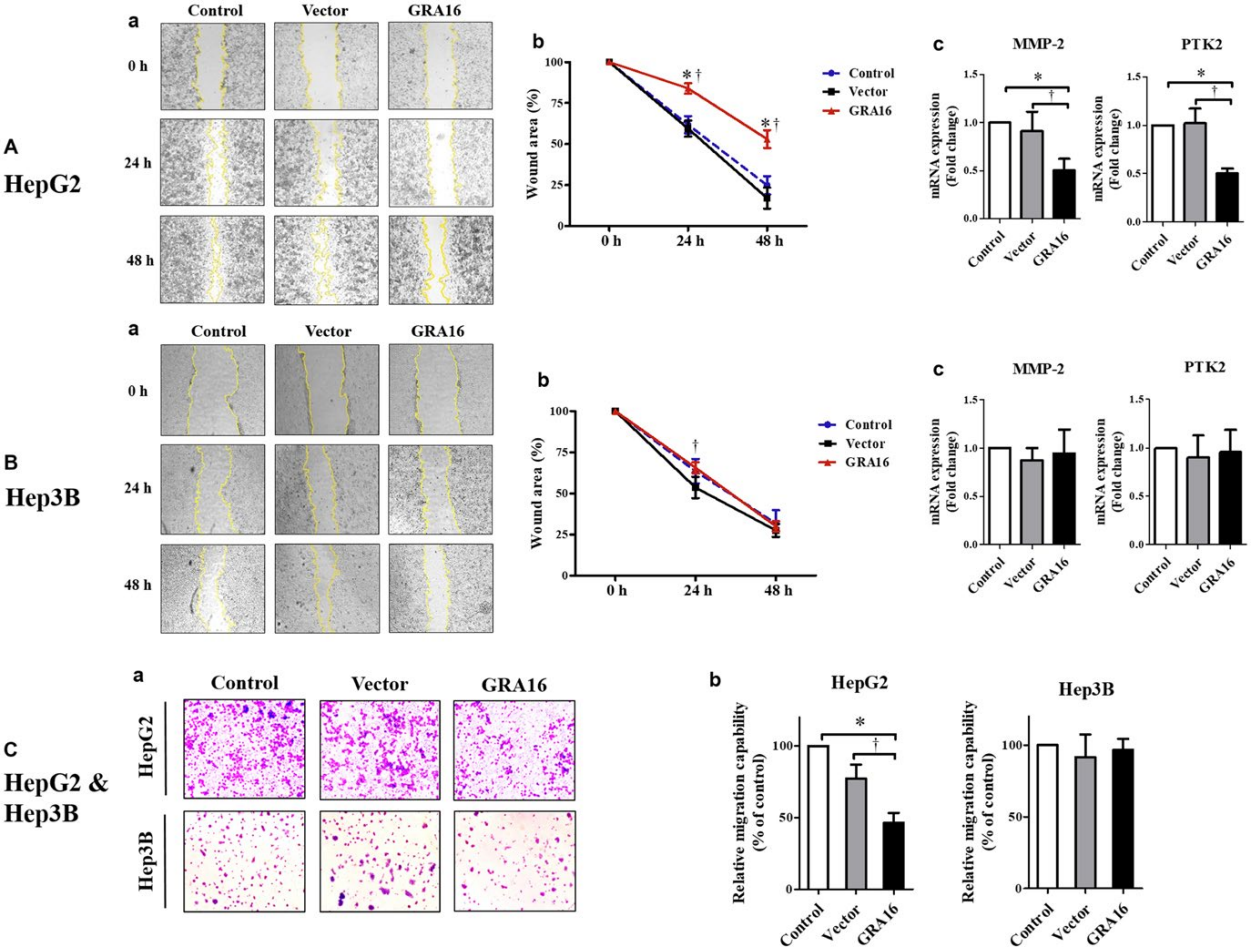
Effects of GRA16 on the cell mobility and invasive activity between HepG2 and Hep3B. (A‐a) and (B‐a) Wound healing assay at 0, 24 and 48 h after scratching on the bottom of the culture plate. (A‐b) and (B‐b) Wounded area (%) calculated by the ImageJ program. (A‐c) and (B‐c) Changes in mRNA levels of migration factors, MMP‐2 and PTK2, in HepG2 and Hep3B. (C‐a) Giemsa‐stained images for the invasive cells at 24 h after incubation in the Transwell invasion assay. (C‐b) Relative invasion ability (%) of the vector and GRA groups compared with the control (100%). *The significant difference between the control and GRA groups (*P* < 0.05). †The significant difference between the vector and GRA groups (*P* < 0.05)

We investigated the changes in the cell mobility (wound closure effect) and invasive activity (Transwell cell migration activity) of cancer cells in HepG2 and Hep3B (Figure 6A‐a and b, B‐a and b and C‐a). In addition, we assessed the mRNA levels of MMP2 and PTK2 (important cell migration factors), which can be increased by AKT phosphorylation and cell mobility factor, which is inhibited by PTEN respectively (Figure 6A‐c and B‐c). In HepG2 cells, the presence of GRA16 significantly reduced cell mobility at 24 and 48 hours after incubation in comparison to the control and vector groups (*P* < 0.05; Figure 6A‐b). In addition, the decreased cell mobility suggests that the wounded area was not reduced (Figure 6A‐a). However, in Hep3B, the wounded area (%) after scratching was covered in the same degree among the control, vector and GRA16 groups, indicating no effect of GRA16 (Figure 6B‐a and b); this result emphasized repeatedly the significance of the endogenous p53 for the anticancer activity of GRA16. Similarly, the mRNA levels of MMP‐2 and PTK2 were significantly reduced in GRA16‐stable HepG2 cells in comparison to control and vector (*P* < 0.05; Figure 6A‐c), but they were not reduced in GRA16‐stable Hep3B cells (Figure 6B‐c). Moreover, the Transwell invasion assay exhibited similar results as follows (Figure 6C‐a). When the result was calculated by the relative migration capability (%) compared with the control (100%), it was also consistent with that of the wound healing assay (Figure 6C‐b). Hence, we are convinced that the effect of GRA16 as a HAUSP inhibitor could induce the anticancer effect in p53‐wild HCC cells.

### 3.7 Tumour reduction in xenograft mice

**Figure 7:**
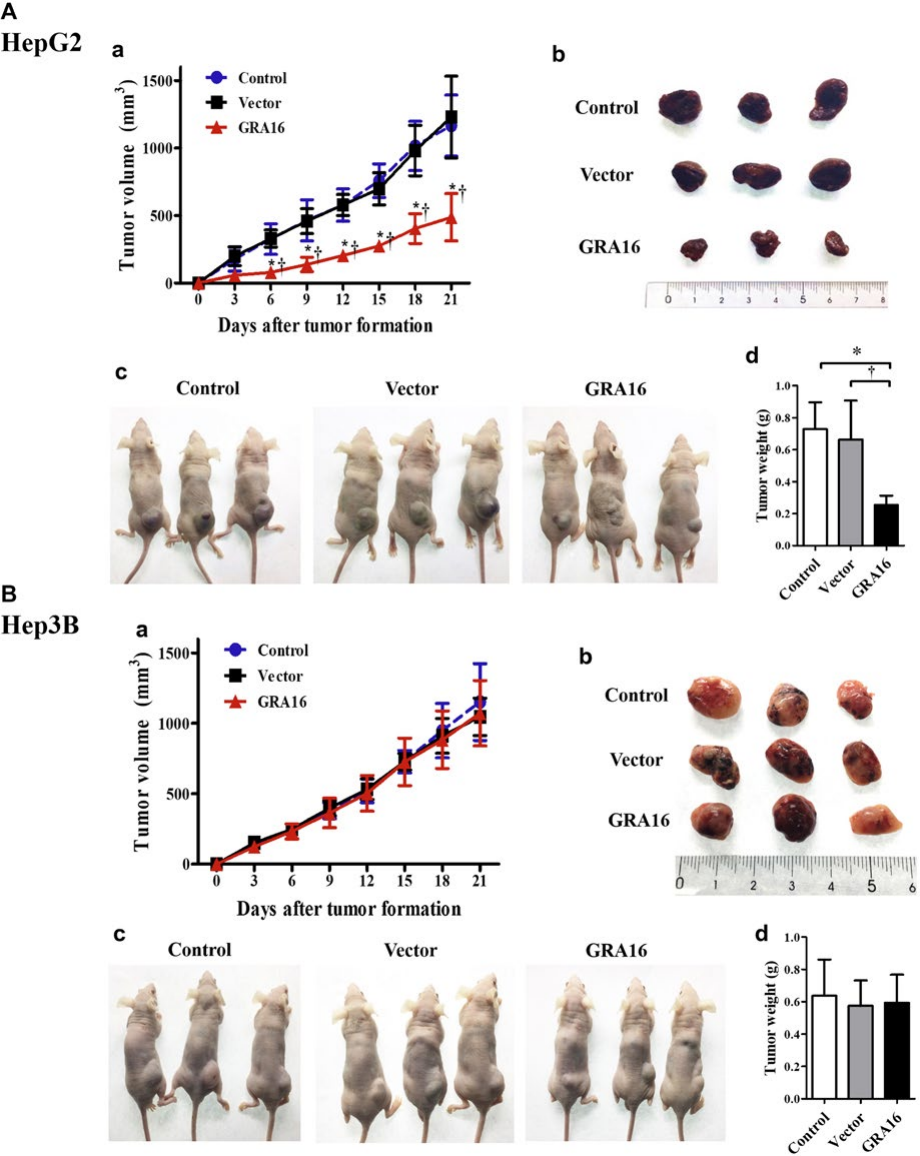
Tumour reduction induced by GRA16 in xenograft mice. Nude mice were used as xenograft model mice. (A‐a and B‐a) The tumour volume (mm^3^) of mice injected with control‐, vector‐ and GRA16‐stable cells in HepG2 and Hep3B. (A‐b and B‐b) Tumour mass isolated by each nude mice. (A‐c and B‐c) Tumour‐bearing mice after the HepG2 or Hep3B cancer cell implantation. (A‐d and B‐d) Weight (g) of the isolated tumour mass. *Significant differences in tumour volume (mm^3^) and tumour weight (g) between the control and GRA groups (*P* < 0.05). †Significant differences in the tumour volume (mm^3^) and tumour weight (g) between the vector and GRA groups (*P* < 0.05)

Nude mice xenograft with control‐ and vector‐HepG2 or Hep3B cells exhibited a gradual increase in the tumour size and mass (Figure 7A‐a and d and B‐a and d). However, the tumour size and mass in nude mice xenograft with GRA‐stable HepG2 cells were significantly lower than that in the control and vector group (*P* < 0.05; Figure 7A‐a and A‐b). The tumour weights in nude mice xenograft with GRA16‐stable HepG2 cells were significantly smaller than those in the control and vector group (*P* < 0.05; Figure 7A‐d). However, the tumour mass and tumour weights in nude mice xenograft with GRA‐stable Hep3B cells were continuously increased without differences in comparison to the control and vector group (Figure 7B‐a and B‐d). Hence, we are convinced that GRA16 in nude mice xenograft enhanced anticancer effects in the presence of p53.

## 4 DISCUSSION

The inhibition of USP (or HAUSP) has been reported as an anticancer drug target.^1^ The action of HAUSP is highly specific for substrates such as MDM2, p53, PTEN and FOXO4.^1^, ^3^, ^8^ Typically, HAUSP is overexpressed in cancer, and its level is highly useful to anticipate the prognosis of the cancer treatment.^1^, ^2^ Previously, various roles of HAUSP inhibitors have been investigated from various angles of the HAUSP/MDM2/p53, HAUSP/PTEN/p53 and HAUSP/p53‐axes, which regulate the transcriptional levels of molecules switching between the survival and death of cells.^1^, ^2^ Thus, our study assessed the vital detailed mechanisms wherein GRA16 as a HAUSP inhibitor acts at the stage of autoregulatory networks of HAUSP/MDM2/PTEN/p53 for inducing anticancer activity.

A high expression of HAUSP is frequent in HCC tissues, and a high HAUSP level correlates with larger tumour size, emphasizing that targeting HAUSP for HCC therapy is clinically imperative, and it is worth investigating HCC suppression using a HAUSP inhibitor.^1^, ^2^, ^3^, ^6^ Typically, GRA proteins of *T gondii* secreted from parasites reside in the parasitophorous vacuole and play a role in the intracellular survival and replication of parasites.^13^ Of these, GRA16 migrates to the nucleus and participates in the regulation of the p53 oncogene signalling pathway.^13^ We assessed whether an anticancer effect could be induced by using the HAUSP‐binding effect of GRA16 in HCC, and, moreover, the underlying mechanisms inducing p53 stabilization after HAUSP inhibition. As some human cancer types, including HCC, exhibit an abnormal *p53* gene or have disrupted *p53* gene activation pathways, the effect of GRA16 should be evaluated in conditions with and without the *p53* gene.^17^ Thus, in our study, we developed genetically modified GRA16‐stable cancer cells for p53‐wild‐type HepG2 and p53‐null‐type Hep3B, and examined the binding between GRA16 and HAUSP within cells using the co‐IP. However, Hep3B cells did not exhibit any changes in the levels of MDM2 and PTEN within cells expressing GRA16. This finding could be construed as debatable owing to the existence of conflicting results for Hep3B cells, for example, HAUSP‐knockdown using siRNA inhibited cell proliferation in Hep3B, and HAUSP inhibition using p5091 also induced apoptosis and cell growth arrest in p53‐mutated lymphocytic leukaemia cell line MEC‐1.^7^, ^8^ The study did not consider the detailed mechanism between HAUSP inhibition and reduced cell proliferation capacity^7^ and observed an increase in the PTEN nuclear pool without further investigating the apoptosis mechanism.^8^ Conversely, other studies reported that the tumour‐suppressor PTEN directly interacted with p53 through the increase of endogenous p53 by deubiquitination and acetylation of p53 in an AKT‐independent manner in hereditary cancer.^6^, ^21^ Other factors inducing apoptosis of Hep3B in in vitro experiments using HepG2 and Hep3B were under the following situation by which p21, p27, p73 and Bax/Bcl‐2 were increased by the action of an MDM2 antagonist or by which AKT inhibition was observed by notch1 overexpression,^22^, ^23^ indicating that the precise mechanisms should be determined by the type of HAUSP inhibitor and the target substrate for the HAUSP inhibitor.

The p53 protein cooperates with PTEN and could be an essential blockage in the development of mammary tumours^6^; thus, we can comprehend that the therapeutic efficiency of anticancer agents strongly depends on their detailed mechanisms to trigger apoptosis in targeted cancer cells.^24^ As p53 plays a pivotal role in regulating tumour apoptosis, our study elucidated the prominence of the p53‐dependent mechanism on the anti‐HCC effect of GRA16. In HCC tumours, down‐regulation of the nuclear PTEN is an essential step in hepatocarcinogenesis.^5^, ^19^, ^20^ As revealed in this study, PTEN levels in deleted p53‐bearing Hep3B cells had decreased in comparison to p53‐wild HepG2 cells. In GRA16‐expressing stable HepG2, apoptotic molecules, expression and nuclear localization of PTEN, endogenous p53 expression, retention of the G_2_‐M phase of the cell cycle and apoptotic cells were increased; however, antiapoptotic molecules, AKT phosphorylation and migration and invasion capacity of HepG2 cells were decreased. These findings strongly suggested that the major mechanism of GRA16 in inducing apoptosis of tumour cells is p53 stabilization according to increase in the nuclear localization of PTEN.

Phosphatase and tensin homologue induces apoptosis of tumours not only by p53 stabilization through its nuclear localization but also by antagonization to AKT phosphorylation.^25^ Thus, PTEN increased by cancer therapy is a crucial down‐regulator of AKT function by AKT dephosphorylation and inhibits cell migration and causes cell cycle arrest in tumour cells.^19^ p53 activation can cause down‐regulation of the cellular AKT level.^26^ Consequently, a decrease in AKT phosphorylation, which is demonstrated in this study, appears to strengthen the antitumor activity in the situation of the autoregulatory feedback between nuclear PTEN and p53, meanwhile, suggesting the importance of the PTEN‐p53‐AKT loop for the anticancer activity of GRA16.^26^ Corroborating a prior study,^8^ the effect of GRA16 in inducing cell cycle arrest resulted in the increase of the G_2_‐M phase and the apoptotic pathway in a PTEN‐dependent manner. Our study presents the new possibility of the existence of an autoregulatory feedback loop between PTEN and p53 in GRA16‐stable HepG2. In particular, because crucial cancer therapy is based on either chemotherapy or radiotherapy, which induces p53‐dependent apoptosis, the presence of wild‐type p53 in tumour cells is the basis for effective chemotherapy.^17^ Accordingly, we emphasize the efficacy of GRA16 in the presence of wild p53 as well as the biology of a specific tumour type. Furthermore, we suggest that GRA16 serves as a complement in increasing the efficacy of other chemotherapeutic agents, although the efficacy of GRA16 could not fully cover the treatment of deleted p53‐bearing HCC. Hence, further studies are required to explore a new HAUSP inhibitor. This study defines the role of GRA16 as a PTEN regulator and highlights the importance of endogenous p53 for GRA16 as a HAUSP inhibitor to induce anticancer effects in both in vitro and in vivo studies, raising the possibility of GRA16 as new supplementary anticancer therapeutics. Furthermore, this study newly proves that the major role of GRA16 is to inhibit PTEN translocation from the nucleus to the cytoplasm through HAUSP inhibition and suggests that GRA16 can be applied as an alternative treatment for HCC.

## CONFLICT OF INTEREST

The authors have no competing interests to declare.

## AUTHOR CONTRIBUTIONS

SGK, SHS and EHS conceived and designed the experiments. SGK and SHS prepared the GRA16 stable cell line and performed the experiments. JHS, JPY and EHS analysed the data. SHL and EHS contributed reagents/materials/analysis tools. EHS supported the idea and was responsible for overall project administration, acquiring financial support and writing the paper.
